# Anthraquinone-Loaded Liposomes for TAM Reprogramming in Triple-Negative Breast Cancer: Mechanistic Rationale, Delivery Logic, and Translational Challenges

**DOI:** 10.3390/pharmaceutics18070781

**Published:** 2026-06-26

**Authors:** Limin Zhai, Juan Liu, Lizhen Mu, Cuiping Li, Siyuan Zhao, Ting Li, Qiuzhen Zhu, Xiaoli Hou, Kourong Shi, Wei Fan

**Affiliations:** Innovative Chinese Medicine Research Institute, Department of Pharmacy, Shanghai Seventh People’s Hospital Affiliated to Shanghai University of Traditional Chinese Medicine, Shanghai 200137, China; liminzhai127@163.com (L.Z.); liujuan2012@163.com (J.L.); lizhensu0714@gmail.com (L.M.); cuiping-li@foxmail.com (C.L.); zsy1045476406@163.com (S.Z.); jwen_tang@126.com (T.L.); zhuqiuzhen@126.com (Q.Z.); 18801790902@139.com (X.H.)

**Keywords:** triple-negative breast cancer, tumor-associated macrophages, anthraquinones, tumor immune microenvironment, liposomes, TAM reprogramming, immunomodulation

## Abstract

Triple-negative breast cancer (TNBC) is an aggressive subtype characterized by limited actionable targets, early recurrence, metastatic propensity, and variable responses to immune checkpoint blockade. Therapeutic resistance is closely associated with myeloid immunosuppression, in which tumor-associated macrophages (TAMs) promote T-cell exclusion, stromal remodeling, angiogenesis, metabolic dysfunction, and resistance to cytotoxic and immune-based therapies. Anthraquinone compounds, including emodin, aloe-emodin, rhein, and chrysophanol, may support TAM reprogramming by regulating tumor-cell stress responses, endoplasmic reticulum stress, immunogenic cell death-associated signaling, redox balance, immunometabolism, and STAT3/NF-κB-related inflammatory pathways. However, poor aqueous solubility, heterogeneous biodistribution, unstable systemic exposure, and potential off-target toxicity limit their translational development. Liposomal delivery offers a formulation strategy to improve solubilization, biodistribution, TAM-associated uptake/engagement, intracellular release, and therapeutic exposure windows. This review discusses anthraquinone-loaded liposomes for TAM reprogramming in TNBC by integrating mechanistic rationale, evidence boundaries, delivery logic, formulation determinants, and translational challenges, with particular attention to stress chaperone proteins, lipid composition, vesicle lamellarity, membrane phase state, responsive release, clinically relevant liposomal formulations, and clinical developability. Overall, anthraquinone-loaded liposomes are better positioned as immune microenvironment recalibration platforms or synergistic modulators in combination therapy rather than as standalone cytotoxic agents for TNBC.

## 1. Introduction

### 1.1. Advances in TNBC Immunotherapy and Unmet Needs

Triple-negative breast cancer (TNBC) is defined by the absence of estrogen receptor, progesterone receptor, and human epidermal growth factor receptor 2 expression [1]. Compared with other breast cancer subtypes, TNBC shows greater aggressiveness, earlier recurrence, higher metastatic potential, and fewer actionable molecular targets [1,2]. Although chemotherapy, antibody–drug conjugates, PARP inhibitors, and immune checkpoint inhibitors have expanded treatment options for selected patients, durable responses remain limited by tumor heterogeneity, immune escape, and therapy-induced resistance [3,4,5].

The heterogeneous benefit of immunotherapy indicates that TNBC treatment response is not determined solely by effector T-cell activation [4,6]. Therapeutic efficacy also depends on whether the tumor immune microenvironment can be shifted from an immunosuppressive state toward an immune-permissive state [6,7,8]. Therefore, strategies that attenuate upstream immunosuppressive networks may improve immune microenvironment recalibration and enhance TNBC responsiveness to combination immunotherapy [4,8,9]. Recent reviews published in 2026 further highlight that TNBC immunotherapy remains limited by tumor heterogeneity, immune-excluded microenvironments, adaptive resistance, and the need for rational combination strategies targeting both tumor-intrinsic vulnerabilities and the tumor immune microenvironment [10,11].

### 1.2. TAMs as Upstream Intervention Nodes for TNBC Immune Microenvironment Remodeling

Tumor-associated macrophages (TAMs) are central myeloid regulators of the TNBC immune microenvironment, linking cytokine-mediated immune suppression, immunometabolic dysregulation, stromal remodeling, angiogenesis, and T-cell dysfunction [12,13,14]. By limiting antigen presentation, secreting immunosuppressive mediators, expressing immune checkpoint ligands, altering metabolic conditions, and restricting effector immune-cell infiltration, TAMs can impair antitumor immunity [13,15,16].

Accordingly, simply enhancing T-cell activity or blocking a single checkpoint may be insufficient in TNBC with dominant myeloid suppression [4,14,15]. Compared with macrophage depletion, TAM reprogramming aims to shift macrophages from tumor-supportive states toward immune-supportive phenotypes while preserving innate immune functions [17,18,19]. This makes TAMs an upstream intervention node for immune microenvironment recalibration and subsequent immunotherapy [9,13,14].

### 1.3. Pharmacological Basis for Anthraquinone-Mediated TAM Reprogramming

Anthraquinones are natural and semisynthetic compounds characterized by a conserved anthraquinone scaffold and diverse substituent patterns [20,21,22]. Representative compounds, including emodin, aloe-emodin, rhein, chrysophanol, and related derivatives, can influence cellular stress responses, oxidative stress, inflammatory signaling, immunometabolism, and tumor–immune interactions [23,24,25].

Among these compounds, emodin provides the most relevant evidence for macrophage regulation and tumor immune microenvironment recalibration, whereas other anthraquinones mainly provide supportive evidence through tumor-cell stress responses, anti-inflammatory effects, or signaling regulation [26,27,28]. However, direct validation of stable TAM reprogramming in TNBC models remains limited, and many conclusions are derived from tumor-cell systems, non-tumor inflammatory models, or cross-model inference [26,27,28,29,30,31]. Thus, anthraquinones should be regarded as mechanistically plausible but not yet fully validated candidates for TAM-oriented intervention.

### 1.4. The Value of Liposomal Delivery Systems in Establishing an Effective Exposure Window

The translational potential of anthraquinones depends on whether their in vivo exposure can be controlled within therapeutically relevant immune niches [31,32,33]. Many anthraquinones suffer from poor aqueous solubility, uneven biodistribution, rapid clearance, and dose-related toxicity, which may limit stable immune modulation in vivo [31,34,35].

Liposomal delivery provides a formulation strategy to improve anthraquinone solubilization, circulation stability, biodistribution, cellular uptake, and intracellular release [31,36,37]. In TNBC, liposomes may facilitate TAM-associated uptake/engagement because macrophages can internalize nanoscale lipid carriers [38,39,40]. However, this should be interpreted as macrophage-associated uptake within the tumor microenvironment rather than absolute TAM-specific targeting [38,39,40]. Its effectiveness remains constrained by tumor heterogeneity, stromal barriers, protein corona formation, immune clearance, and release behavior [33,41,42,43].

### 1.5. Aim and Scope of This Review

**Figure 1:**
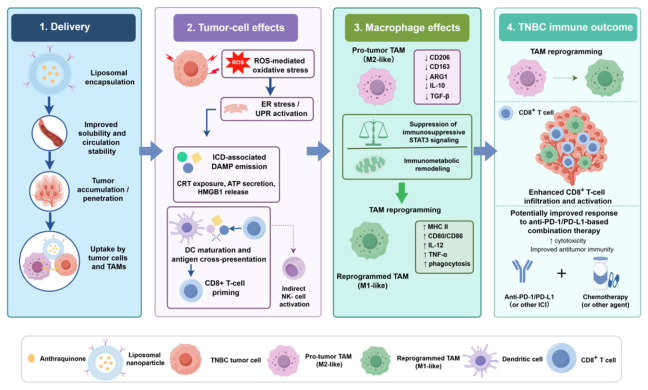
Proposed role of anthraquinone-loaded liposomes in TAM reprogramming and immune remodeling in TNBC. In the marker boxes, downward arrows indicate decreased expression of pro-tumor/M2-associated markers, including CD206, CD163, ARG1, IL-10, and TGF-β, whereas upward arrows indicate increased expression or activity of reprogrammed/M1-associated markers and functions, including MHC II, CD80/CD86, IL-12, TNF-α, and phagocytosis. Created with FigDraw (www.figdraw.com; authorization code: IWWAO42233).

This review evaluates anthraquinone-loaded liposomes as a potential strategy for TAM reprogramming in TNBC [13,31,38]. It focuses on the biological rationale for TAM intervention, the pharmacological basis and evidence boundaries of anthraquinone-mediated immune modulation, the delivery logic and formulation determinants of liposomal systems, and the translational challenges that must be addressed before clinical development [13,31,33]. Rather than presenting anthraquinone-loaded liposomes as established TNBC therapeutics, this review emphasizes their potential role as immune microenvironment recalibration platforms or synergistic modulators in combination therapy. To directly illustrate the proposed role of anthraquinone-loaded liposomes in TNBC, a schematic overview is provided in Figure 1, highlighting liposomal delivery, tumor-cell stress/ICD induction, TAM reprogramming, and downstream CD8^+^ T-cell-mediated immune remodeling.

## 2. Literature Search Strategy and Evidence Mapping

This review was designed as a narrative, mechanistic, and translational review rather than a formal systematic review or meta-analysis. To improve the transparency and reproducibility of evidence identification, selection, and organization, a structured literature search and an adapted PRISMA-style evidence-mapping workflow were performed to identify publications related to anthraquinone compounds, liposomal delivery systems, tumor-associated macrophages (TAMs), and triple-negative breast cancer (TNBC). The adapted PRISMA-style workflow was used only to facilitate transparent evidence mapping and was not intended for quantitative synthesis or formal risk-of-bias assessment.

PubMed, Web of Science, Scopus, and Google Scholar were searched for publications available up to June 2026. To ensure adequate coverage of the most recent literature, particular attention was given to publications published in 2026 related to TNBC-associated TAMs, macrophage reprogramming, anthraquinone pharmacology, emodin/aloe-emodin, liposomal delivery systems, nanomedicine-enabled immune modulation, clinically established liposomal formulations, and translational challenges.

The search strategy combined disease-related, immune-related, compound-related, and formulation-related terms. Representative search terms included “triple-negative breast cancer”, “TNBC”, “tumor-associated macrophages”, “TAMs”, “TAM reprogramming”, “macrophage polarization”, “anthraquinones”, “emodin”, “aloe-emodin”, “rhein”, “chrysophanol”, “liposomes”, “liposomal delivery”, “nanomedicine”, “drug delivery”, “immunomodulation”, “tumor immune microenvironment”, “endoplasmic reticulum stress”, “immunogenic cell death”, “STAT3”, “NF-κB”, “stress chaperone proteins”, and “immunometabolism”. Representative Boolean combinations included “TNBC AND tumor-associated macrophages”, “(anthraquinone OR emodin OR rhein) AND macrophage polarization”, “liposome AND breast cancer AND macrophage”, “anthraquinone AND endoplasmic reticulum stress AND immunogenic cell death”, and “liposomal formulation AND cancer AND clinical translation”. The targeted 2026 update search additionally included terms such as “2026”, “macrophages next-generation cancer immunotherapy”, “senescence-like TAMs”, “IL-6/STAT3”, “lipid–polymer hybrid liposomes”, “cell membrane-coated liposomes”, “nanomaterial-enabled TAM modulation”, and “liposomal nanotherapeutics cancer immunotherapy”.

Additional relevant publications were identified through manual screening of reference lists from selected original articles and reviews. Publications were included when they provided mechanistic, pharmacological, formulation-related, immunological, or translational information relevant to TNBC immune heterogeneity, TAM-mediated immunosuppression, anthraquinone pharmacology, macrophage polarization, stress-response signaling, liposomal drug delivery, lipid composition, vesicle structure, intracellular release, nanomedicine-enabled immune modulation, or clinically relevant liposomal anticancer formulations. Publications were excluded when they were unrelated to cancer or immune regulation, lacked mechanistic or formulation relevance, provided insufficient information for evaluating anthraquinone pharmacology or liposomal delivery, or consisted of duplicate records, editorials, conference abstracts without accessible full-text data, or non-English publications lacking sufficient accessible information.

The initial database search identified 386 records. After duplicate removal, title/abstract screening, full-text eligibility assessment, and exclusion of ineligible full-text articles, 164 unique publications were included in the initial narrative evidence map. The targeted update search up to June 2026 identified 14 additional eligible publications. After duplicated references were counted only once, 178 unique publications were included in the revised narrative review. The adapted PRISMA-style evidence-mapping workflow is summarized in Supplementary Figure S1.

## 3. Biological Roles of Tumor-Associated Macrophages in the TNBC Immune Microenvironment

### 3.1. TNBC Immune Heterogeneity and TAM Accumulation

The immune microenvironment of triple-negative breast cancer (TNBC) is highly heterogeneous across patients and even among different regions within the same tumor [7,44,45]. Although TNBC generally exhibits higher immunogenicity than some other breast cancer subtypes, this immunogenic potential is frequently counterbalanced by multilayered immunosuppressive mechanisms, including myeloid cell accumulation, impaired effector T-cell infiltration, metabolic restriction, abnormal angiogenesis, extracellular matrix remodeling, and immune checkpoint activation [2,6,7,16]. The limited clinical benefit of immune checkpoint blockade in many patients indicates that immune resistance in TNBC is not solely caused by insufficient antigenicity or inadequate T-cell activation, but rather reflects a broader imbalance within the tumor immune ecosystem [4,7,8,46]. Consistently, recent reviews published in 2026 on the breast cancer/TNBC tumor microenvironment further emphasize that rational immunotherapy should account for cellular, metabolic, stromal, and structural complexity rather than focusing solely on tumor-intrinsic vulnerabilities or effector T-cell activation [47,48].

Within this suppressive ecosystem, tumor-associated macrophages (TAMs) represent one of the most important myeloid populations [13,14,49]. TAMs connect cytokine-mediated immune suppression, metabolic remodeling, angiogenesis, extracellular matrix deposition, and T-cell exclusion [14,16,50]. Their abundance and functional state are closely associated with tumor progression, metastasis, therapeutic resistance, and poor prognosis [13,51,52,53]. Therefore, TAMs provide a biologically rational entry point for immune microenvironment recalibration in TNBC [13,17,54]. Consistently, a 2026 systematic review of TAM-targeted therapies in TNBC further reinforces the view that TAMs are not only orchestrators of the tumor microenvironment but also actionable therapeutic targets [55].

TAMs in TNBC are mainly derived from circulating monocytes recruited into tumor tissues, although tissue-resident macrophages may also contribute to the intratumoral macrophage pool [13,51,56]. Their recruitment, differentiation, and functional polarization are regulated by tumor- and stromal-derived signals, including CCL2–CCR2, CSF-1–CSF1R, hypoxia-related factors, inflammatory cytokines, lactate, and extracellular matrix-derived cues [12,50,57,58]. After entering the tumor microenvironment, macrophages are continuously shaped by local stress signals and intercellular communication [44,56,57,59].

Importantly, TAM phenotypes should not be interpreted according to a rigid M1/M2 binary classification [18,60,61]. Instead, they represent a dynamic and heterogeneous continuum [56,60,61]. In TNBC, many TAM subsets display M2-like or immunosuppressive features, including increased expression of CD206, CD163, ARG1, IL-10, TGF-β, VEGF, and PD-L1 [13,14,49,62]. However, their degree of immunosuppression, antigen-presenting capacity, metabolic status, and reprogrammability may vary substantially among hypoxic regions, perivascular areas, invasive margins, necrotic regions, and stromal-rich niches [44,45,56,63]. This spatial and functional heterogeneity is highly relevant to therapeutic design because TAM-targeted strategies may be effective only when macrophages remain functionally plastic and serve as dominant regulatory nodes within the local immune microenvironment [17,18,54,64].

### 3.2. Mechanisms of TAM-Mediated Immunosuppression

TAMs suppress antitumor immunity in TNBC through multiple interconnected mechanisms rather than through a single dominant pathway [13,14,49]. One major mechanism involves the establishment of immunosuppressive cytokine networks [14,65,66]. IL-10 can impair antigen presentation and reduce effector immune activation, whereas TGF-β promotes immune tolerance, suppresses cytotoxic lymphocyte function, and contributes to stromal remodeling [66,67,68]. VEGF not only promotes angiogenesis but also contributes to vascular abnormality and impaired immune-cell infiltration [2,7,69]. Together, these mediators reinforce a suppressive and structurally restrictive microenvironment [7,14,16,70].

Key signaling pathways further sustain TAM-mediated immunosuppression [71,72,73]. STAT3 is a central regulator of immunosuppressive macrophage programming and promotes the expression of IL-10, ARG1, PD-L1, VEGF, and other suppressive mediators [71,72,74]. NF-κB signaling is more context-dependent; depending on its activation intensity, duration, and cellular context, it may participate in inflammatory activation or chronic tumor-promoting inflammation [73,75,76]. Thus, therapeutic modulation of these pathways should be understood as rebalancing signaling outputs rather than simply inhibiting STAT3 or NF-κB activity [72,73,74,76].

TAMs also contribute to metabolic and structural immune suppression [14,77,78]. They participate in lactate accumulation, extracellular acidification, nutrient restriction, oxidative stress adaptation, and lipid metabolic remodeling [50,77,79,80]. These metabolic changes impair T-cell activation, proliferation, cytotoxic function, and persistence [81,82,83]. In parallel, TAMs promote extracellular matrix deposition, collagen remodeling, abnormal angiogenesis, and stromal barrier formation, thereby restricting immune-cell trafficking and generating immune-excluded tumor regions [14,16,70]. Therefore, TAM-mediated immunosuppression in TNBC reflects an integrated network of cytokine signaling, immune checkpoint expression, metabolic restriction, vascular abnormality, and tissue-structure remodeling [13,14,57].

TAMs also interact extensively with tumor cells, stromal cells, endothelial cells, dendritic cells, myeloid-derived suppressor cells, and T lymphocytes [57,59,84]. Through these interactions, they function as integrators of innate immune regulation and adaptive immune suppression [7,57,84]. Tumor cells can recruit and educate macrophages through chemokines, growth factors, metabolic products, and stress-associated signals [12,50,57]. In return, TAMs support tumor progression by promoting angiogenesis, matrix remodeling, immune tolerance, and metastatic dissemination [13,14,51].

The interaction between TAMs and T cells is particularly relevant to TNBC immunotherapy [7,85,86]. TAMs can restrict CD8^+^ T-cell infiltration through stromal remodeling and vascular dysfunction, while also impairing T-cell activation through IL-10, TGF-β, PD-L1, metabolic competition, and antigen-presentation defects [14,15,16,68]. Even when immune checkpoint inhibitors partially restore T-cell activity, adaptive immune responses may remain weak or transient if TAM-mediated myeloid suppression and spatial exclusion are not relieved [4,15,46,85]. Therefore, TAMs should be viewed as network-level regulators that influence whether antitumor immunity can be initiated, amplified, and maintained [17,18,54,57].

### 3.3. Rationale for TAM Reprogramming

Given the central role of TAMs in TNBC-associated immune suppression, TAM reprogramming has emerged as an upstream strategy for immune microenvironment recalibration [13,14,54]. This rationale is further supported by the recent literature published in 2026, including a TNBC-focused systematic review of TAM-targeted therapies and a broader Cancer Cell article identifying macrophages as key targets for next-generation cancer immunotherapy [55,87]. Unlike macrophage depletion, which may remove both protumor and potentially beneficial macrophage populations, reprogramming aims to shift TAMs from an immunosuppressive and tumor-supportive state toward a more immune-supportive phenotype while preserving innate immune functions [17,18,19,64]. This approach may improve antigen presentation, restore phagocytic activity, reduce suppressive cytokine output, enhance inflammatory and chemokine signaling, and facilitate CD8^+^ T-cell infiltration [38,54,88,89].

The objective of TAM reprogramming is not simply to induce transient pro-inflammatory activation [18,54,64]. Excessive or poorly controlled macrophage activation may increase nonspecific inflammatory perturbation and reduce therapeutic tolerability [9,54,90]. A more appropriate goal is to recalibrate the immune microenvironment by reducing suppressive network redundancy while preserving immune balance [8,9,54]. In line with this view, recent perspectives published in 2026 on macrophage plasticity emphasize that precision-targeted tumor immunotherapy should exploit macrophage functional plasticity while avoiding uncontrolled inflammatory activation or nonspecific macrophage depletion [91]. This requires sustained, spatially appropriate, and dose-controlled modulation of macrophage function [92,93,94].

This requirement is particularly important for small-molecule immunomodulators such as anthraquinones [23,24,34]. Their immunological effects may depend strongly on exposure level, exposure duration, intracellular distribution, and local microenvironmental context [31,32,33,34]. Therefore, TAM reprogramming provides not only a biological rationale for anthraquinone-based intervention but also a formulation rationale for delivery systems capable of controlling biodistribution, cellular uptake, and intracellular release [31,88,94,95]. This provides the conceptual basis for discussing anthraquinone-mediated TAM reprogramming and liposome-enabled exposure-window control in the following sections.

Recent evidence further supports the relevance of TAM plasticity in TNBC therapy resistance. Zhou et al. reported that chemotherapy can induce senescence-like TAMs in TNBC and that TAM-derived IL-6 promotes drug resistance and cancer stemness through the IL-6/IL-6R/STAT3 axis, highlighting the therapeutic importance of targeting macrophage-associated adaptive resistance [96].

## 4. Pharmacological Basis and Evidence Boundaries of Anthraquinone-Mediated TAM Reprogramming

### 4.1. Representative Anthraquinones and Evidence Positioning

Anthraquinones are natural or semisynthetic compounds characterized by a conserved quinone-containing aromatic scaffold and diverse substituent patterns [20,21,22]. Representative members include emodin, aloe-emodin, rhein, chrysophanol, physcion, sennoside-related compounds, and their derivatives [20,29,30,97,98]. Their redox activity, hydrophobicity, membrane interaction, and pharmaceutical modifiability provide a chemical basis for regulating oxidative stress, mitochondrial function, endoplasmic reticulum stress, inflammatory signaling, and metabolic adaptation [23,24,25,99,100].

Anthraquinones should not be interpreted as a functionally uniform class, because their biological activity, immunological effects, toxicity profile, and formulation suitability depend on substituent pattern, dose, exposure duration, experimental model, and tissue distribution [21,22,29,30,34]. Among representative anthraquinones, emodin provides the strongest TAM-related evidence. It has been reported to regulate macrophage polarization, inflammatory mediator production, NF-κB-, STAT1/IRF5-, STAT6/IRF4-, and STAT3-associated pathways, and tumor–macrophage crosstalk [26,27,28]. In breast cancer-related models, emodin has also been associated with reduced macrophage infiltration and M2-like polarization, enhanced T-cell activation, inhibition of angiogenesis, and attenuation of protumor macrophage feedback [26,27]. Other anthraquinones, including aloe-emodin, rhein, chrysophanol, physcion, and sennoside-related compounds, mainly provide indirect support through oxidative stress regulation, ER stress, inflammatory signaling, apoptosis, antigen presentation, or adaptive immune activation [23,29,30,98]. Therefore, emodin should be considered the lead anthraquinone candidate for TAM-oriented investigation, whereas other anthraquinones mainly support the mechanistic plausibility of this strategy. Recent reviews on emodin and aloe-emodin further emphasize that anthraquinone pharmacology is characterized by multitarget regulation, redox and inflammatory signaling modulation, poor bioavailability, potential toxicity, and the need for delivery-enabled translational strategies [22,29].

### 4.2. Tumor-Cell Stress, ERS/ICD Signaling, and DAMP Emission

Anthraquinones may influence the tumor immune microenvironment through stress-associated immunogenic signaling in addition to direct tumor-cell cytotoxicity [100,101,102]. By inducing ROS generation, mitochondrial dysfunction, ER stress, UPR activation, and regulated tumor-cell death, anthraquinones may promote ICD-associated DAMP emission, including calreticulin exposure, ATP secretion, and HMGB1 release [34,100,102,103,104]. These signals can be sensed by dendritic cells, macrophages, and other innate immune cells, thereby linking tumor-cell injury with antigen presentation and adaptive immune activation [102,103,104].

Stress chaperone proteins, including HSP90, HSP70, HSP27, and GRP78/BiP, may further connect anthraquinone-induced proteostatic stress with immune remodeling [105,106,107]. However, the tumor-cell stress–ERS/UPR–ICD/DAMP–stress chaperone axis should be interpreted as an upstream immunogenic input rather than direct proof of TAM reprogramming [77,101,102,103]. Further studies are needed to determine whether anthraquinone-loaded liposomes modulate this axis in TNBC models and whether such changes correlate with macrophage phenotype switching, antigen presentation, CD8^+^ T-cell infiltration, and antitumor efficacy [31,54,100,103].

### 4.3. Macrophage-Intrinsic Regulation

In addition to tumor-cell-derived signals, anthraquinones may directly affect macrophage-intrinsic programs related to mitochondrial activity, redox balance, glycolysis, lipid metabolism, cytokine output, and transcriptional signaling [23,28,77,79]. Available evidence suggests that emodin can modulate macrophage polarization, inflammatory mediator release, and lipid metabolism-related pathways, with reported involvement of NF-κB-, STAT1/IRF5-, STAT6/IRF4-, EGFR/MAPK-, and IL-6/JAK2/STAT3-associated signaling in macrophage- or tumor-related models [26,27,28,108,109,110].

STAT3 and NF-κB are particularly relevant to TAM regulation [71,72,73]. Sustained STAT3 activation supports immunosuppressive macrophage programming and promotes IL-10, ARG1, PD-L1, VEGF, and related suppressive mediators [71,72,74,111]. NF-κB signaling is more context-dependent and may contribute either to inflammatory activation or chronic tumor-promoting inflammation depending on activation intensity, duration, and microenvironmental context [73,75,76,112]. Therefore, anthraquinone-mediated regulation of these pathways should be described as signaling rebalancing rather than simple pathway inhibition [23,28,72,112].

Importantly, changes in ROS, metabolism, STAT3, or NF-κB alone do not prove stable TAM reprogramming in TNBC [54,64,71,113]. Functional validation should include macrophage-specific and spatially resolved endpoints, such as CD86, MHC II, iNOS, CD206, CD163, ARG1, IL-10, TGF-β, phagocytosis, antigen-presentation capacity, TAM uptake/engagement, intracellular anthraquinone exposure, and spatial association with CD8^+^ T cells [31,32,54,64,85].

### 4.4. Evidence Hierarchy and Key Gaps

The current evidence supporting anthraquinone-mediated TAM reprogramming can be divided into four evidence levels [54,64,113]. Direct evidence is mainly associated with emodin and includes regulation of macrophage polarization, tumor–macrophage crosstalk, angiogenesis, T-cell activation, and M2-like macrophage-associated tumor progression [26,27,28]. Indirect evidence comes from anthraquinone-induced tumor-cell stress, ERS/UPR activation, ICD-associated DAMP emission, inflammatory modulation, and stress chaperone-related proteostatic disturbance [100,101,102,103,104,105]. Inferential evidence is based on pathway overlap, including redox balance, mitochondrial function, glycolysis, lipid metabolism, STAT3, NF-κB, and cytokine networks relevant to TAM biology [71,72,73,77,79,80,112].

Several gaps remain unresolved. First, high-quality evidence demonstrating stable anthraquinone-mediated TAM reprogramming in TNBC models remains limited [26,27,28,31,54,64]. Second, anthraquinone-mediated immune effects are highly dependent on dose, exposure duration, tissue distribution, and intracellular localization [31,33,34,35]. Third, tissue-level accumulation does not necessarily indicate sufficient intracellular exposure in TAMs [31,32,33,36,41]. Fourth, the stress chaperone–ICD–TAM axis remains mechanistically plausible but incompletely validated [101,102,103,104,105].

Overall, anthraquinones, particularly emodin, possess pharmacological features compatible with TAM-oriented immune modulation [23,26,27,28]. A recent 2026 review on emodin and the anthraquinone scaffold further underscores the discrepancy between multitarget pharmacological promise and current translational limitations, including poor bioavailability, potential toxicity, and the need for delivery-enabled translational strategies [22]. However, anthraquinone-loaded liposomes should be positioned as delivery-enabled immunomodulatory platforms or combination-therapy sensitizers rather than as established standalone TAM-reprogramming therapeutics [31,32,33,36,54,64,95]. The mechanistic levels, evidence types, and research maturity are summarized in Table 1.

## 5. Delivery Logic and Formulation Determinants of Anthraquinone-Loaded Liposomes

### 5.1. Delivery Cascade and Effective Exposure Window

Although anthraquinones have demonstrated antitumor and immunomodulatory potential in vitro and in animal models, their direct in vivo application remains limited by poor aqueous solubility, unstable systemic exposure, heterogeneous biodistribution, rapid clearance, and dose-related toxicity [29,31,34,35]. These limitations are particularly relevant for TAM reprogramming because immune modulation generally requires sustained and controlled exposure rather than transient high peak concentrations [33,92,95]. Insufficient exposure may fail to induce stable macrophage functional reorientation, whereas excessive exposure may cause nonspecific cytotoxicity, inflammatory perturbation, or organ toxicity [31,33,35,54].

Liposomal delivery provides a formulation strategy to address these limitations [31,37,115]. Consistently, a recent 2026 review of emodin-based drug delivery systems further emphasizes that nanocarrier platforms, including liposomes, may help overcome emodin-related translational barriers such as poor aqueous solubility, limited bioavailability, rapid systemic elimination, and toxicity-related concerns [116]. Hydrophobic anthraquinones can be incorporated into lipid bilayers, while lipid composition, cholesterol content, particle size, surface charge, PEGylation, vesicle lamellarity, membrane phase state, and release behavior can be adjusted to regulate circulation stability, tissue distribution, protein corona formation, cellular uptake, and intracellular drug release [31,36,37,42,117]. Thus, liposomes function not only as solubility- or tumor-accumulation-enhancing carriers, but also as exposure-engineering platforms capable of shaping biodistribution, TAM-associated uptake/engagement, intracellular release, and immune-compatible exposure windows [31,32,33,36,38,95].

The delivery process of anthraquinone-loaded liposomes can be conceptualized as a cascade involving systemic circulation, tumor access, intratumoral distribution, TAM-associated uptake/engagement, and intracellular release [31,36,38,41]. At the tissue level, liposomes may accumulate in tumors through enhanced vascular permeability and impaired lymphatic drainage [43,118]. However, the enhanced permeability and retention effect is highly heterogeneous across tumor types, patients, and intratumoral regions and should be regarded as a permissive condition for tumor entry rather than a reliable targeting mechanism [33,41,43,118].

After reaching TNBC tissues, liposomes must penetrate the extracellular matrix, diffuse within the interstitial space, and access TAM-enriched immune niches [32,33,38,41]. Abnormal vasculature, high interstitial pressure, dense extracellular matrix, hypoxia, and necrotic regions may restrict nanocarrier distribution [32,41,43,63]. Consequently, total tumor accumulation does not necessarily indicate sufficient exposure within TAM-associated niches [33,36,41,42]. Spatial distribution should therefore be evaluated when possible, particularly whether liposomes colocalize with macrophage-rich areas, perivascular regions, or invasive fronts [33,38,39,41].

At the cellular level, TAMs are highly phagocytic and may preferentially internalize liposomes within the tumor microenvironment [38,39,119]. This provides a biological basis for TAM-oriented delivery, although macrophage uptake is influenced by particle size, surface charge, PEGylation, lipid composition, protein corona formation, and local macrophage phenotype [37,38,42,117]. Protein corona formation may further reshape liposome–macrophage interactions [42,117]. Therefore, TAM uptake should be validated in relevant tumor models rather than inferred solely from particle size or in vitro macrophage internalization data [33,38,39,41].

Most importantly, cellular uptake does not automatically translate into functional reprogramming [33,36,38,54]. After internalization, anthraquinones must be released from liposomal bilayers or intracellular compartments at sufficient concentrations and for appropriate durations to modulate macrophage signaling, metabolism, and phenotype [31,36,71,77,95]. Evaluation of anthraquinone-loaded liposomes should therefore extend beyond tissue accumulation and include intracellular exposure, release kinetics, macrophage-specific endpoints, downstream immune responses, and safety boundaries [31,33,36,41,54,64]. Only when these steps form a coherent delivery-to-function chain can liposomal delivery be interpreted as enabling TAM reprogramming [31,33,36,38,54].

### 5.2. Lipid Composition and Membrane Engineering

The chemical composition of lipids is a fundamental determinant of liposome performance [37,115,120]. For anthraquinone-loaded liposomes, lipid composition affects drug-loading efficiency, membrane retention, circulation stability, protein corona formation, macrophage uptake, release kinetics, storage stability, and toxicity [31,37,40,42,117,120]. Therefore, formulation design should be discussed at the level of specific lipid components rather than treating liposomes as generic carriers [37,120].

Phospholipids constitute the structural basis of the liposomal bilayer [37,121]. Commonly used phospholipids include hydrogenated soy phosphatidylcholine, distearoylphosphatidylcholine, dipalmitoylphosphatidylcholine, egg phosphatidylcholine, and soy phosphatidylcholine [37,120,121]. Saturated phospholipids with high phase-transition temperatures generally form more rigid and stable membranes, reducing premature leakage and supporting stability during systemic circulation [37,120,121]. In contrast, unsaturated phospholipids form more fluid membranes, which may facilitate drug release but increase instability during storage or systemic circulation [37,120,121]. For hydrophobic anthraquinones, phospholipid selection directly affects bilayer compatibility, encapsulation efficiency, membrane retention, and release behavior [31,37,95,120].

Cholesterol is another key component of many liposomal formulations [37,121]. By inserting between phospholipid molecules, cholesterol regulates membrane packing, reduces membrane permeability, improves physical stability, and decreases premature leakage [37,120,121]. However, its effect is concentration-dependent [37,120]. Appropriate cholesterol content stabilizes the bilayer and improves drug retention, whereas excessive cholesterol may restrict membrane fluidity or alter bilayer organization, reduce drug-loading capacity, or interfere with controlled release [37,95,120,121]. For anthraquinone-loaded liposomes, cholesterol optimization is particularly important because many anthraquinones are expected to partition within or near the hydrophobic bilayer domain [31,37,120].

PEGylated lipids, such as DSPE-PEG2000, are commonly introduced to prolong systemic circulation and reduce mononuclear phagocyte system clearance [37,41,117]. PEGylation improves colloidal stability and decreases nonspecific protein adsorption, but excessive PEG density may reduce cellular interactions and limit TAM uptake [37,40,42,117]. This creates a formulation dilemma for TAM-oriented delivery: prolonged circulation favors tumor access, whereas sufficient carrier–cell interaction is required for macrophage uptake [38,39,41,117]. Therefore, PEGylation should balance stealth behavior and TAM-associated internalization rather than be maximized indiscriminately [37,40,42,117].

Charged lipids further modulate liposome–cell interactions [37,121,122]. Cationic lipids may enhance membrane binding and cellular uptake, but they are often associated with stronger serum protein adsorption, faster immune clearance, complement activation, and higher systemic toxicity [37,40,42,117,122]. Anionic or mildly charged liposomes may show better tolerability and more controllable biodistribution, although uptake efficiency varies with the biological environment [37,41,42,122]. Neutral liposomes are generally less reactive but may require additional strategies to enhance intracellular delivery [36,37]. Thus, surface charge should be selected according to the intended balance among stability, macrophage uptake, systemic safety, and translational feasibility [31,36,37,38,41,122].

Overall, lipid composition determines whether anthraquinone-loaded liposomes maintain sufficient stability before reaching tumor tissues while allowing TAM-associated uptake/engagement and intracellular release within TAM-associated niches [31,36,37,38,41,95]. Future studies should report lipid species, lipid molar ratios, cholesterol content, PEG-lipid density, charged lipid content, encapsulation efficiency, drug-to-lipid ratio, leakage rate, serum stability, and storage stability to support formulation reproducibility and translational interpretation [33,37,115,120,123].

### 5.3. Supramolecular Structure: Lamellarity and Membrane Phase State

In addition to lipid composition, the supramolecular structure of liposomes influences drug loading, release behavior, biodistribution, tumor accumulation, and cellular uptake [37,41,120,121]. Liposomes may exist as small unilamellar vesicles, large unilamellar vesicles, or multilamellar vesicles [37,121]. These forms differ in bilayer number, aqueous core volume, membrane surface area, drug localization, and release kinetics and should be considered when designing anthraquinone-loaded systems [31,37,120,121].

Unilamellar liposomes contain a single phospholipid bilayer and are generally more uniform in size when prepared by extrusion, microfluidics, or other controlled manufacturing methods [37,115,121]. They may support more reproducible circulation and tissue-distribution behavior, which is advantageous for systemic delivery [37,41,115]. However, for hydrophobic anthraquinones, drug-loading capacity depends mainly on bilayer volume and lipid–drug compatibility [31,37]. Thus, unilamellar vesicles with limited bilayer content may provide lower hydrophobic drug-loading capacity than multilamellar systems unless lipid composition is carefully optimized [37,121].

Multilamellar liposomes contain multiple concentric lipid bilayers and may provide larger hydrophobic domains for lipophilic anthraquinones [31,37,121]. This structure may increase drug retention and prolong release, but it may also cause larger particle size, broader size distribution, reduced tumor penetration, and greater batch-to-batch variability [33,37,41,43,115]. Because tumor accumulation and TAM-associated uptake/engagement are strongly influenced by particle size and colloidal uniformity, multilamellar structures require strict control in TAM-oriented delivery [37,38,39,41,43].

The physical state of the lipid membrane is another important factor [37,120,121]. Liposomal bilayers may exist in a liquid-crystalline or gel-like state depending on phospholipid composition, cholesterol content, and temperature relative to the lipid phase-transition temperature [37,120,121]. Liquid-crystalline membranes are more fluid and may facilitate drug diffusion and release, but excessive fluidity can increase premature leakage during circulation [37,95,120,121]. Gel-like membranes are more ordered and rigid, improving storage stability and reducing leakage but potentially slowing drug release and reducing intracellular availability after TAM uptake [36,37,95,120,121]. Therefore, membrane phase behavior should match the intended release window and immunomodulatory objective [36,37,95,120].

For anthraquinone-loaded liposomes, membrane state is particularly important because hydrophobic anthraquinones are expected to partition with the acyl-chain region of the bilayer [31,37,120]. Changes in membrane fluidity, packing density, and phase-transition behavior influence drug partitioning, retention, and release [37,120,121]. A rigid bilayer may improve systemic stability but delay intracellular release, whereas a more fluid bilayer may enhance release but increase premature leakage and off-target exposure [36,37,95,120,121]. Optimal formulation design should therefore balance extracellular stability with timely intracellular release after TAM-associated uptake/engagement [36,37,38,95].

**Figure 2:**
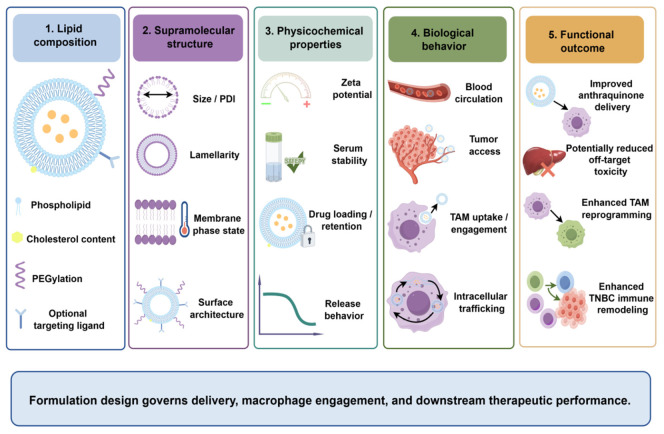
Formulation determinants governing anthraquinone-loaded liposome performance and downstream TAM-oriented immune modulation. Note: Lipid composition, supramolecular structure, and physicochemical properties collectively influence circulation behavior, tumor access, intratumoral distribution, TAM-associated uptake/engagement, intracellular trafficking, and drug release. These factors ultimately determine anthraquinone exposure within TAM-associated niches and may influence TAM reprogramming, downstream immune remodeling, and therapeutic responses in TNBC. Created with FigDraw (www.figdraw.com; authorization code: UIPAAb55ee).

These structural considerations also affect tumor accumulation [37,41,43]. Particle size, lamellarity, membrane rigidity, and surface properties influence circulation half-life, extravasation, tumor penetration, protein corona formation, mononuclear phagocyte system clearance, and TAM-associated uptake/engagement [37,38,41,42,43,117]. Therefore, the molecular and supramolecular structure of anthraquinone-loaded liposomes should be evaluated together with conventional physicochemical parameters, including particle size, polydispersity index, zeta potential, morphology, encapsulation efficiency, drug release, serum stability, and storage stability [33,37,115,120]. Such characterization is essential for linking formulation properties to biological performance and translational feasibility [33,37,115,120]. These interrelated formulation determinants and their downstream implications for TAM-oriented immune modulation are summarized in Figure 2.

### 5.4. Responsive Release and Intracellular Exposure

Responsive release strategies are used to improve the spatial and temporal precision of liposomal drug delivery [36,95,124]. In tumors, acidic pH, elevated reactive oxygen species, high glutathione levels, and increased protease activity may serve as endogenous triggers for pH-responsive, redox-responsive, or enzyme-responsive liposomes [114,124,125,126]. These systems are designed to remain stable during circulation while accelerating drug release in tumor tissues, intracellular compartments, or TAM-associated niches [36,95,127,128].

For anthraquinone-loaded liposomes, responsive release should not be judged only by faster in vitro release [33,36,95,129]. The key issue is whether the trigger exists at sufficient intensity in the target microenvironment and improves intracellular anthraquinone exposure in relevant cell populations [32,33,36,41,114]. pH-responsive systems may promote release in acidic endosomes or lysosomes after macrophage uptake, whereas redox-responsive systems may exploit intracellular redox gradients to facilitate cytoplasmic drug release [127,128,130,131]. Enzyme-responsive systems may be useful in matrix metalloproteinase- or protease-enriched tumor regions, but only when enzyme activity overlaps spatially with TAM-associated niches [114,124,132].

Responsive design should therefore be considered a tool for exposure-window control rather than an independent advantage [33,36,95,114]. A useful responsive system should meet three criteria: biological relevance of the trigger in TNBC or TAM-associated compartments; increased intracellular effective exposure rather than nonspecific drug leakage; and translation into measurable immunological endpoints, such as reduced CD206/CD163 expression, increased antigen-presentation markers, enhanced phagocytosis, decreased IL-10/TGF-β output, increased CD8^+^ T-cell infiltration, or improved immunotherapy response [38,54,64,85,88].

Accordingly, anthraquinone-loaded liposomes should be evaluated by linking release behavior with biological function [31,33,36,88,95]. In vitro release profiles, serum stability, endosomal escape, intracellular localization, TAM-associated uptake/engagement, phenotypic reprogramming, and downstream immune activation should be analyzed together [36,37,38,88,95,115,128]. This integrated evaluation is necessary to determine whether responsive release improves TAM reprogramming rather than simply adding formulation complexity [33,36,88,124]. Recent reviews published in 2026 further emphasize that advanced liposomal systems, including lipid–polymer hybrid liposomes, cell membrane-coated liposomes, surface-functionalized liposomes, and stimuli-responsive liposomes, may improve formulation stability, targeting performance, and controlled release; however, their clinical translation remains constrained by manufacturing scalability, long-term stability, immune interactions, and safety evaluation [133,134]. The key formulation determinants that should be considered when designing anthraquinone-loaded liposomes for TAM-oriented delivery are summarized in Table 2.

### 5.5. Marketed and Clinically Established Liposomal Formulations

Although no liposomal formulation has been developed specifically for anthraquinone-mediated TAM reprogramming in TNBC, marketed and clinically established liposomal products provide important translational references for formulation design, exposure control, toxicity mitigation, co-delivery, and immune modulation. These formulations should not be interpreted as direct TNBC-specific evidence for anthraquinone-loaded liposomes. Instead, they illustrate how liposomal platforms can reshape biodistribution, improve the clinical usability of poorly soluble or poorly tolerated drugs, support combination therapy, and create formulation-dependent therapeutic exposure windows [115,133,134,135,136,137,138,139,140].

Representative examples include PEGylated liposomal doxorubicin (Doxil/Caelyx), non-PEGylated liposomal doxorubicin (Myocet), paclitaxel liposome formulations such as Lipusu, liposomal irinotecan (Onivyde), fixed-ratio daunorubicin/cytarabine liposomes (Vyxeos), and the liposomal immunomodulator mifamurtide (Mepact) [133,136,137,138,139,140]. Together, these products demonstrate that clinically established liposomal formulations depend not only on the encapsulated drug but also on lipid composition, particle size, lamellarity, PEGylation, drug retention, release kinetics, manufacturability, safety, and rational clinical positioning [37,115,133,134,135,136,137,138,139,140]. These lessons are directly relevant to the development of anthraquinone-loaded liposomes as delivery-enabled immunomodulatory platforms for TAM engagement and TNBC immune remodeling. These translational lessons are summarized in Table 3.

## 6. Mechanistic Framework of Anthraquinone-Loaded Liposomes in TNBC

### 6.1. Proposed Delivery-Enabled Mechanism for TAM Reprogramming

The proposed mechanism of anthraquinone-loaded liposomes in TNBC can be understood as a delivery-enabled immune microenvironment remodeling process rather than a simple enhancement of cytotoxic drug exposure [31,32,33,36,38,95]. After systemic administration, liposomal encapsulation may improve the solubility, circulation stability, and tumor-access probability of hydrophobic anthraquinones [31,37,41,43,95,115,121,135]. Within TNBC tissues, liposomes may accumulate partly through vascular permeability-related mechanisms and may be internalized by phagocytic myeloid cells, including TAMs [38,39,40,41,43,118,119,144]. This process provides a formulation basis for increasing the probability of TAM-associated exposure within TAM-associated niches, although such enrichment should be interpreted as relative exposure bias rather than absolute macrophage targeting [38,40,41,42,43,117].

After TAM-associated uptake/engagement and intracellular release, anthraquinones may influence both tumor-cell stress responses and macrophage-intrinsic programs [31,36,38,95]. In tumor cells, anthraquinones may induce oxidative stress, mitochondrial dysfunction, endoplasmic reticulum stress, unfolded protein response activation, stress chaperone responses, and immunogenic cell death-related signaling [99,100,101,102,105,145]. These events may promote the exposure or release of damage-associated molecular patterns, including calreticulin, ATP, and HMGB1, thereby providing immunogenic inputs to macrophages and antigen-presenting cells [102,103,104]. However, the final immune outcome depends on the timing, intensity, and spatial context of stress signaling, as well as the functional state of surrounding myeloid cells [54,56,64,146,147,148].

In TAMs, anthraquinones may modulate macrophage-intrinsic pathways associated with immunometabolism, redox balance, STAT3 signaling, NF-κB output, and inflammatory mediator production [23,28,71,72,77,79,112]. These effects should be evaluated by immunosuppressive markers, including IL-10, TGF-β, ARG1, VEGF, PD-L1, CD206, and CD163, together with functional endpoints such as antigen presentation, phagocytosis, inflammatory signaling, and T-cell recruitment [27,28,54,64,88]. Through tumor-cell stress induction, macrophage-intrinsic rebalancing, and adaptive immune amplification, anthraquinone-loaded liposomes may promote a shift from an immunosuppressive TAM state toward a more immune-supportive phenotype [38,54,64,85,88].

Therefore, the therapeutic value of anthraquinone-loaded liposomes should be evaluated not only by tumor growth inhibition, but also by whether delivery and release translate into measurable macrophage and adaptive immune endpoints [31,38,54,64,88,95]. Relevant endpoints include TAM phenotype, antigen-presentation markers, phagocytic activity, cytokine profiles, CD8^+^ T-cell infiltration, T-cell effector function, and responsiveness to combination immunotherapy [38,54,64,85,88].

### 6.2. Functional Positioning in Combination Therapy

Given their proposed role in alleviating myeloid immunosuppression, anthraquinone-loaded liposomes are more appropriately positioned as immune microenvironment-calibrating agents or synergistic modulators rather than standalone cytotoxic therapies [31,38,54,64,88]. Their clinical value is likely to depend on whether TAM reprogramming can improve the initial immune conditions required for subsequent immune amplification [54,64,85,89]. In this context, anthraquinone-loaded liposomes may function as preconditioning agents that convert a myeloid-suppressed or immune-excluded tumor microenvironment into a more permissive state for immunotherapy [8,54,64,89,149].

A rational combination strategy is integration with immune checkpoint blockade, particularly PD-1/PD-L1-targeted therapy [4,15,46]. In TNBC, limited response to checkpoint inhibitors is often associated with inadequate T-cell infiltration, T-cell exhaustion, myeloid suppression, and stromal exclusion [4,7,15,46,85]. If anthraquinone-loaded liposomes can reduce TAM-mediated suppression, enhance antigen presentation, and improve CD8^+^ T-cell accessibility, they may increase the probability and durability of response to immune checkpoint blockade [15,38,54,64,85].

Other potential combinations include innate immune agonists, chemotherapy, radiotherapy, photodynamic therapy, and immunometabolic modulators [126,150,151,152,153]. These approaches may increase tumor antigen release, induce immunogenic stress, activate dendritic cells or macrophages, normalize vascular or stromal barriers, or relieve metabolic constraints on immune cells [103,150,151,152,153]. However, combination development should follow the principle of minimal effective complexity: each added therapeutic component or formulation module should demonstrate a clear contribution to delivery behavior, TAM functional endpoints, downstream T-cell responses, or safety improvement [54,64,123,154,155,156]. Clinically, anthraquinone-loaded liposomes may be most plausible as preconditioning or synergistic platforms in selected TNBC subgroups characterized by high TAM infiltration, dominant myeloid suppression, and retained immune plasticity [2,4,45,54,64,132,157,158].

## 7. From Mechanistic Plausibility to Developmental Feasibility: Key Translational Boundaries and Future Directions

### 7.1. Safety Boundaries: Intrinsic Toxicity of Anthraquinones and Risk Management

Safety remains a central translational boundary for anthraquinone-loaded liposomes [31,33,122,159,160,161,162,163]. Anthraquinones may cause toxicity through multiple mechanisms, including redox cycling, oxidative stress, mitochondrial dysfunction, metal ion chelation, DNA damage, and photosensitization [25,35,164,165,166,167]. Although the most systematic evidence for cardiotoxicity is derived mainly from anthracycline and anthraquinone-like anticancer drugs, these findings provide useful mechanistic guidance for risk identification [136,168,169,170]. Natural anthraquinones should therefore not be assumed to be free of cardiovascular, hepatic, mitochondrial, or phototoxic risks [35,164,165,166,167,168,169,170]. Instead, safety boundaries should be defined on a compound-specific and formulation-specific basis [33,37,42,115,122].

Liposomal delivery may mitigate certain toxicity risks by reducing the fraction of free drug, smoothing peak plasma concentrations, altering tissue distribution, and limiting nonspecific exposure [37,115,135,136,137,138]. However, liposomes do not eliminate the intrinsic toxicity of anthraquinones; rather, they redistribute and reshape exposure-related risks [31,33,37,115]. Repeated dosing, cumulative tissue retention, mononuclear phagocyte system uptake, and long-term organ exposure may introduce new safety concerns [33,40,41,122]. In addition, liposomal properties such as lipid composition, surface charge, PEGylation, particle size, lamellarity, membrane phase state, and release kinetics can influence complement activation, macrophage accumulation, organ distribution, and systemic tolerability [37,40,41,42,122,159,162].

Therefore, safety evaluation should not rely only on short-term tumor inhibition or single-dose toxicity studies [33,122,129]. A translationally relevant safety framework should integrate pharmacokinetic exposure, tissue distribution, cardiac function, hepatic and renal biomarkers, hematological parameters, inflammatory cytokines, phototoxicity, mitochondrial injury, and repeated-dose tolerability [35,41,122,136,167,168,169,170]. For immunomodulatory liposomes, the key question is whether the formulation can support TAM reprogramming within an acceptable exposure window without causing systemic inflammation or organ toxicity [31,33,37,54,64].

### 7.2. Applicability Boundaries: Immune Microenvironment Heterogeneity and Patient Stratification

TNBC is not a uniform disease entity, and its immune microenvironment varies substantially among patients and tumor regions [2,7,44,45,132,171,172]. TAM abundance, spatial distribution, phenotypic composition, suppressive pathway activity, vascular permeability, stromal density, hypoxia, and interstitial pressure may all influence the therapeutic performance of anthraquinone-loaded liposomes [13,41,43,44,45,56,63]. Therefore, this strategy should not be assumed to be broadly applicable to all TNBC cases [2,4,44,45,132,157,158].

The most likely responsive population may include tumors characterized by high TAM infiltration, myeloid-dominated immunosuppression, active suppressive signaling pathways such as IL-6/JAK/STAT3, and sufficient delivery accessibility [2,13,14,54,111,132,157,158]. In contrast, tumors with low TAM abundance, T-cell suppression driven mainly by non-myeloid mechanisms, or highly restrictive stromal and vascular barriers may respond poorly to TAM-oriented liposomal delivery [2,4,41,43,56,63]. Thus, therapeutic suitability should be defined by both biological relevance and delivery feasibility [33,41,43,54,64,132,157,158].

Future studies should integrate patient stratification into study design [132,157,158]. Potential stratification indicators include TAM burden, CD206/CD163-positive macrophage distribution, MHC II expression, IL-10/TGF-β/ARG1/PD-L1 expression, STAT3 pathway activity, CD8^+^ T-cell infiltration, hypoxia status, vascular permeability, stromal density, and imaging-based nanocarrier accessibility [2,13,41,44,45,54,63,111,132,157,158]. Only by combining immune phenotyping with delivery-accessibility assessment can anthraquinone-loaded liposomes be developed as a precision immunomodulatory strategy rather than a population-averaged intervention [33,41,43,54,64,132,157,158].

### 7.3. Evidence Boundaries: Efficacy Evaluation Frameworks and Biomarker Development

For anthraquinone-loaded liposomes, conventional cytotoxic endpoints such as tumor volume reduction are insufficient to define therapeutic value [31,33,54,64]. Because the proposed mechanism involves immune microenvironment remodeling, efficacy evaluation should be based on a mechanism-informed and temporally resolved framework [38,54,64,88,95]. The administered dose alone is not an adequate metric; instead, the relationship among biodistribution, intracellular exposure, TAM functional changes, downstream T-cell responses, and safety should be assessed [31,33,36,41,54,64,85,95].

A rational evaluation framework should include at least four layers. The first layer is delivery confirmation, including tumor accumulation, intratumoral distribution, TAM colocalization, macrophage uptake, and intracellular release [31,33,36,38,39,41,95]. The second layer is TAM functional assessment, including changes in CD206, CD163, ARG1, IL-10, TGF-β, VEGF, PD-L1, MHC II, CD86, iNOS, phagocytosis, antigen presentation, and inflammatory cytokine output [13,54,64,71]. The third layer is adaptive immune response, including CD8^+^ T-cell infiltration, activation, exhaustion status, granzyme B expression, and spatial proximity between T cells and tumor cells [7,15,85,89]. The fourth layer is net therapeutic benefit, including tumor suppression, recurrence control, metastasis inhibition, survival, inflammatory burden, and organ safety [31,33,54,64,122,168,169,170].

Biomarker development should therefore move beyond single static markers [157,158] Composite biomarkers combining TAM burden, TAM phenotype, suppressive cytokine profiles, STAT3/NF-κB activity, immunometabolic features, CD8^+^ T-cell status, and delivery-accessibility indicators may be more informative [13,44,45,50,54,64,77,79,132,157,158]. Spatial transcriptomics, single-cell sequencing, multiplex immunofluorescence, flow cytometry, and quantitative imaging may help establish whether anthraquinone-loaded liposomes truly convert delivery advantages into TAM reprogramming and downstream immune activation [44,45,85,132,171,172,173].

### 7.4. Manufacturing, Quality Control, and Minimal Effective Complexity

Recent macrophage-centered immunotherapy literature reinforces the view that TAMs are not merely suppressive bystanders but therapeutically actionable immune regulators. However, successful translation requires appropriate patient stratification, rigorous validation of macrophage reprogramming, and rational integration with existing immunotherapy or chemotherapy platforms [54,64,96,133,174]. In parallel, manufacturing feasibility and quality control are critical determinants of whether anthraquinone-loaded liposomes can progress from mechanistic plausibility to translational development [33,115,123,155,156,175]. Although ligand modification, stimulus-responsive release, co-delivery, and multifunctional designs may improve performance in experimental models, each additional module increases formulation complexity, manufacturing variability, quality-control requirements, and regulatory burden [64,123,154,155,156,175]. For liposomal systems, product performance is influenced not only by the encapsulated drug but also by lipid composition, lipid molar ratio, cholesterol content, PEG-lipid density, charged lipid content, drug-to-lipid ratio, encapsulation efficiency, lamellarity, particle size distribution, zeta potential, membrane phase state, drug localization, leakage rate, serum stability, storage stability, and sterilization conditions [37,115,120,121,123].

Therefore, anthraquinone-loaded liposomes should be developed according to the principle of minimal effective complexity [33,123,154,155,156,175]. Initial optimization should focus on fundamental formulation parameters, including lipid composition, particle size, surface charge, cholesterol content, PEGylation, lamellarity, membrane phase state, and release behavior [37,95,115,120,121,123]. Additional targeting ligands, responsive modules, or co-loaded agents should be incorporated only when they provide measurable improvements in delivery efficiency, intracellular exposure, TAM-related endpoints, immune responses, or safety profiles [33,54,64,123,154,155]. Design elements that improve in vitro release characteristics or fluorescence accumulation without demonstrating meaningful biological benefit or translational reproducibility should not be considered sufficient justification for increased formulation complexity [33,36,123,154,156].

Quality by design (QbD) principles should be incorporated early in formulation development [123]. Critical material attributes and critical process parameters should be systematically linked to key product attributes and relevant biological outcomes [33,123,156]. For example, lipid composition and cholesterol content should be associated with membrane stability and drug release; particle size and PDI should be linked to tumor penetration and TAM-associated uptake/engagement; PEGylation and surface charge should be related to circulation behavior, protein corona formation, and immune clearance; and lamellarity and membrane phase state should be connected to hydrophobic drug loading and intracellular availability [36,37,40,41,42,43,117]. Establishing these relationships is essential for scale-up manufacturing, batch-to-batch consistency, regulatory evaluation, and eventual clinical translation [33,123,129,155,156,175].

### 7.5. Future Priorities: From Proof-of-Concept to Development Pathways

Future research should move beyond proof-of-concept studies and establish a continuous evidence chain linking formulation design, delivery behavior, intracellular exposure, immune remodeling, therapeutic benefit, and safety [31,33,36,37,41,54,64]. First, more clinically relevant models should be used, including orthotopic TNBC models, immunocompetent mouse models, metastatic models, patient-derived models, and ex vivo tumor–immune co-culture systems [13,31,44,45,54,64,127]. These models are more suitable than simple subcutaneous models for evaluating whether anthraquinone-loaded liposomes truly engage TAM-associated niches and reshape the immune microenvironment [31,33,38,41,44,45,127].

Second, mechanistic validation should include macrophage-specific and spatially resolved readouts [54,56,64,85]. Future studies should determine whether liposomes are taken up by TAMs in vivo, whether anthraquinones are released intracellularly, whether ERS/ICD-related pathways are modulated, and whether these changes lead to durable TAM reprogramming [31,36,38,54,64,100,103].

Single-cell sequencing, spatial transcriptomics, multiplex immunostaining, flow cytometry, and imaging-based biodistribution analysis may help establish this delivery-to-function relationship [44,45,85,132,171,172,173].

Third, combination strategies should be developed stepwise [33,54,64,154,155]. Anthraquinone-loaded liposomes may be most useful as preconditioning or synergistic agents combined with immune checkpoint inhibitors, STING/TLR agonists, chemotherapy, radiotherapy, photodynamic therapy, or immunometabolic modulators [8,15,126,150,151,152,153]. However, combination regimens should be optimized according to dosing sequence, release kinetics, immune activation window, inflammatory toxicity, and patient stratification [33,54,64,95,157,158].

Finally, pharmaceutical development should be integrated with immunological validation from the early stage [33,54,64,123,129]. Future studies should define critical quality attributes, optimize scalable manufacturing processes, evaluate long-term stability, assess repeated-dose toxicity, and establish regulatory-relevant characterization methods [33,115,122,123,129,176,177,178]. The ultimate development goal should not be the most complex formulation, but a reproducible, interpretable, and scalable system that satisfies four criteria: reliable delivery to relevant niches, genuine TAM reprogramming, verifiable immunological benefit, and translational feasibility [31,33,36,54,64,123,129].

## 8. Conclusions

Triple-negative breast cancer remains difficult to treat because of its aggressive biological behavior, limited molecular targets, and heterogeneous responses to immunotherapy [1,2,3,4]. A major barrier to durable therapeutic benefit is the persistence of a myeloid-dominated immunosuppressive microenvironment, in which tumor-associated macrophages function as central regulators of cytokine-mediated immune suppression, immunometabolic dysregulation, stromal remodeling, angiogenesis, and T-cell exclusion [7,13,14,16]. TAM reprogramming therefore represents an upstream strategy for immune microenvironment recalibration rather than merely enhancing downstream T-cell activation [17,54,64].

Anthraquinone compounds, particularly emodin, provide a mechanistically plausible basis for TAM reprogramming [23,26,27,28]. Their reported effects on endoplasmic reticulum stress, unfolded protein response activation, immunogenic cell death-related signaling, stress chaperone proteins, redox regulation, immunometabolism, STAT3, and NF-κB pathways suggest that they may influence both tumor-cell stress responses and macrophage functional states [23,72,73,100,101,102,103,104,105]. However, current evidence remains uneven. Direct support for stable TAM reprogramming in TNBC is still limited, and many conclusions are derived from tumor-cell models, non-tumor inflammatory systems, or indirect mechanistic inference [26,27,28,31,54,64]. Thus, anthraquinones should be regarded as promising but not yet fully validated candidates for TAM reprogramming.

Liposomal delivery provides a formulation strategy for translating the pharmacological potential of anthraquinones into more controllable in vivo activity [31,33,36,37,115]. By modulating lipid composition, cholesterol content, PEGylation, surface charge, lamellarity, membrane phase state, particle size, biodistribution, cellular uptake, intracellular release, and relative TAM-associated enrichment, liposomes may help establish a controllable exposure window within TAM-associated niches [31,36,37,38,41,95]. The value of anthraquinone-loaded liposomes therefore lies not simply in increasing drug loading or tumor accumulation, but in linking formulation design to intracellular exposure, TAM functional endpoints, downstream T-cell responses, and safety boundaries [31,33,36,54,64,85].

Future development should move beyond proof-of-concept studies and adopt a more rigorous translational framework [33,54,64]. Key priorities include validating TAM-specific uptake and TAM reprogramming in immunocompetent and orthotopic TNBC models, incorporating stress chaperone and immunogenic cell death markers, defining exposure–response relationships, developing composite immune biomarkers, identifying responsive patient subgroups, and establishing scalable manufacturing and quality-control strategies [31,44,45,54,64,102,103,104,105,123,132,157,158]. Overall, anthraquinone-loaded liposomes are best positioned as immune microenvironment recalibration platforms or synergistic modulators in combination therapy rather than as standalone cytotoxic agents for TNBC.

## Figures and Tables

**Table 1:** Evidence hierarchy and validation requirements for anthraquinone-mediated TAM reprogramming.

Evidence Level	Main Mechanistic Basis	Representative Evidence	Current Interpretation	Key Validation Requirements	References
Direct evidence	Macrophage polarization; tumor–macrophage crosstalk; T-cell activation	Emodin-related regulation of macrophage polarization, M2-like features, angiogenesis, and protumor feedback	Strongest support for emodin, but incomplete TNBC-specific validation	TAM phenotype markers; phagocytosis; antigen presentation; TNBC model validation	[26,27,28]
Indirect evidence	Tumor-cell stress; ERS/UPR; ICD-associated DAMP emission; stress chaperones	ROS, ER stress, CRT exposure, ATP secretion, HMGB1 release, and HSP-related stress responses	Upstream immunogenic input, not direct proof of TAM reprogramming	DC activation; macrophage phenotype switching; CD8^+^ T-cell infiltration; antitumor efficacy	[25,102,103,104,105,114]
Inferential evidence	Redox, metabolic, and inflammatory pathway overlap	Mitochondrial function, glycolysis, lipid metabolism, STAT3, NF-κB, and cytokine networks	Mechanistic plausibility, but limited causal evidence	Pathway intervention; spatial immune profiling; rescue experiments	[71,72,77,79,80,112]
Delivery-dependent evidence	Solubilization; biodistribution; TAM uptake/engagement; intracellular release	Improved solubility, circulation behavior, cellular uptake, intracellular release, and potential TAM-associated exposure	Required to convert pharmacological plausibility into immune modulation	Tumor accumulation; TAM colocalization; intracellular release; immune endpoints; safety	[31,32,33,36,37,38,39,95]

Note: TAM, tumor-associated macrophage; TNBC, triple-negative breast cancer; ERS, endoplasmic reticulum stress; UPR, unfolded protein response; ICD, immunogenic cell death; DAMP, damage-associated molecular pattern; CRT, calreticulin; DC, dendritic cell. Evidence levels indicate the relative strength of current support rather than confirmed clinical efficacy.

**Table 2:** Key formulation determinants.

Formulation Determinant	Main Role	Relevance to Anthraquinone Loading/Delivery	TAM-Related Implication	Translational Concern
Particle size/PDI control	Regulates circulation, tumor penetration, and cellular uptake	Influences biodistribution, tumor access, and drug exposure	Affects the probability of TAM-associated uptake/engagement	Requires reproducible manufacturing and stable size distribution
Surface charge modulation	Regulates immune-cell interaction and protein corona formation	Influences serum stability, macrophage interaction, and nonspecific adsorption	May increase macrophage-associated uptake, but excessive charge may cause off-target interactions	Cationic charge may increase toxicity, complement activation, and MPS clearance
Phospholipid type	Determines bilayer fluidity, rigidity, and stability	Affects hydrophobic drug partitioning, membrane retention, and release	Influences stability before TAM uptake and release after internalization	Requires control of lipid source, purity, and phase-transition behavior
Cholesterol content	Regulates membrane packing and permeability	Can reduce premature leakage but may affect loading capacity	Shapes release kinetics and intracellular exposure duration	Excessive cholesterol may delay release or alter membrane behavior
PEGylated lipids	Prolong circulation and reduce nonspecific clearance	Improves colloidal stability and systemic exposure	May reduce premature MPS clearance but also limit cellular internalization	Requires balance between stealth behavior and TAM engagement
Lamellarity	Determines bilayer number and hydrophobic domain volume	Multilamellar vesicles may increase hydrophobic drug loading and retention	May prolong release but reduce tumor penetration if particle size increases	Requires strict control of size, PDI, and batch consistency
Membrane phase state	Controls membrane fluidity and release	Liquid-crystalline membranes may favor release; gel-like membranes may favor retention	Affects intracellular release after macrophage uptake	Must balance systemic stability and intracellular availability
Responsive release module	Controls release in acidic, redox, or enzyme-rich environments	May increase intracellular anthraquinone release	Can enhance TAM exposure if the trigger overlaps with TAM-associated niches	Added complexity must translate into measurable immune benefit

Note: PDI, polydispersity index; TAM, tumor-associated macrophage; MPS, mononuclear phagocyte system. “TAM-associated uptake/engagement” indicates macrophage engagement within the tumor microenvironment and should not be interpreted as absolute TAM-specific targeting.

**Table 3:** Marketed or clinically established liposomal formulations and translational lessons for anthraquinone-loaded liposomes.

Product	Encapsulated Drug	Liposomal Platform/Formulation Feature	Approved or Clinically Established Indication	Relevance to Breast Cancer/TNBC	Translational Lessons for Anthraquinone-Loaded Liposomes	References
Doxil/Caelyx	Doxorubicin hydrochloride	PEGylated liposomal doxorubicin; long-circulating “stealth” liposome	Approved PEGylated liposomal anthracycline formulation for selected malignancies, including metastatic breast cancer in patients at increased cardiac risk, ovarian cancer, multiple myeloma, and AIDS-related Kaposi sarcoma	Not TNBC-specific, but directly relevant as a clinically established anthracycline liposome with breast cancer-related use	Demonstrates that liposomal reformulation can alter anthracycline biodistribution, prolong circulation, and reduce selected free-drug-associated toxicity risks, supporting exposure-window control	[136,137,138]
Myocet	Doxorubicin	Non-PEGylated liposomal doxorubicin	Used in combination with cyclophosphamide for the first-line treatment of metastatic breast cancer in adult women	Directly relevant to metastatic breast cancer management and liposomal anthracycline delivery	Provides a breast cancer-related precedent showing that non-PEGylated liposomal platforms can also be clinically useful when formulation design, dosing, and combination strategy are optimized	[136,137,138]
Lipusu	Paclitaxel	Paclitaxel liposome nanoformulation for hydrophobic drug delivery	Marketed paclitaxel liposome formulation used in solid tumor treatment settings	Relevant to taxane-based breast cancer chemotherapy and hydrophobic anticancer drug loading	Supports the feasibility of liposomal delivery for poorly water-soluble anticancer agents and highlights the importance of drug loading, membrane retention, release behavior, and tolerability	[139]
Onivyde	Irinotecan	PEGylated liposomal irinotecan formulation	Approved for metastatic pancreatic adenocarcinoma in combination regimens, including first-line NALIRIFOX and post-gemcitabine fluorouracil/leucovorin-based therapy	Not breast cancer-specific, but relevant as a clinically established liposomal formulation for exposure modulation	Illustrates the clinical value of liposomal control of pharmacokinetics, tissue exposure, and combination therapy, which is relevant to anthraquinone-loaded liposomes as exposure-engineering platforms	[141]
Vyxeos	Daunorubicin + cytarabine	Fixed-ratio dual-drug liposomal co-formulation	Approved for adults with newly diagnosed therapy-related AML or AML with myelodysplasia-related changes	Not breast cancer-specific, but relevant to rational liposomal co-delivery	Demonstrates that liposomes can maintain defined drug ratios in vivo, supporting future co-delivery concepts involving anthraquinones with chemotherapy, immune checkpoint blockade, or macrophage-modulating agents	[142]
Mepact	Mifamurtide	Liposomal immunomodulator based on muramyl tripeptide phosphatidylethanolamine	EU-approved with postoperative chemotherapy for non-metastatic high-grade osteosarcoma	Not breast cancer-specific, but mechanistically relevant to macrophage/innate immune modulation	Provides a clinically established precedent for liposome-enabled immune modulation, supporting the concept that liposomal formulations can be developed not only for cytotoxic delivery but also for myeloid immune engagement	[143]

Note: These formulations do not directly validate anthraquinone-loaded liposomes for TNBC or TAM reprogramming. They are included as translational precedents showing how liposomal design can influence solubilization, circulation behavior, biodistribution, drug retention, toxicity profile, co-delivery feasibility, immune engagement, manufacturability, and clinical positioning. TAM, tumor-associated macrophage; TNBC, triple-negative breast cancer.

## Data Availability

No new data were created or analyzed in this study. Data sharing is not applicable to this article.

## Supplementary Materials

The following supporting information can be downloaded at: https://www.mdpi.com/article/10.3390/pharmaceutics18070781/s1. Supplementary Figure S1. Adapted PRISMA-based literature screening and targeted update workflow. A total of 386 records were initially identified from PubMed, Web of Science, Scopus, and Google Scholar. After screening and eligibility assessment, 164 unique publications were included in the initial narrative evidence map. Fourteen additional eligible publications were incorporated through a targeted update search up to June 2026. After duplicated references were counted only once, 178 unique publications were included in the revised narrative review.

## Abbreviations

TNBC: Triple-negative breast cancer; TAMs: Tumor-associated macrophages; ERS: Endoplasmic reticulum stress; UPR: Unfolded protein response; ICD: Immunogenic cell death; DAMPs: Damage-associated molecular patterns; MPS: Mononuclear phagocyte system; ROS: Reactive oxygen species; NF-κB: Nuclear factor kappa B; STAT3: Signal transducer and activator of transcription 3; TGF-β: Transforming growth factor beta; PD-L1: Programmed death-ligand 1; QbD: Quality by design.
