# Supplementary material for: Anthraquinone-Loaded Liposomes for TAM Reprogramming in Triple-Negative Breast Cancer: Mechanistic Rationale, Delivery Logic, and Translational Challenges
Source: Pharmaceutics. 2026 Jun 26;18(7):781. doi: 10.3390/pharmaceutics18070781 (PMC13414993; doi:10.3390/pharmaceutics18070781)
Supplement: Supplementary file 1 [file pharmaceutics-18-00781-s001.zip › pharmaceutics-4274206-supplementary-done.pdf]

pharmaceutics

MDPI

Supplementary Material: Anthraquinone-Loaded Liposomes for TAM Reprogramming in Triple-Negative Breast Cancer: Mechanistic Rationale, Delivery Logic, and Translational Challenges

Limin Zhai, Juan Liu, Lizhen Mu, Cuiping Li, Siyuan Zhao, Ting Li, Qiuzhen Zhu, Xiaoli Hou, Kourong Shi and Wei Fan

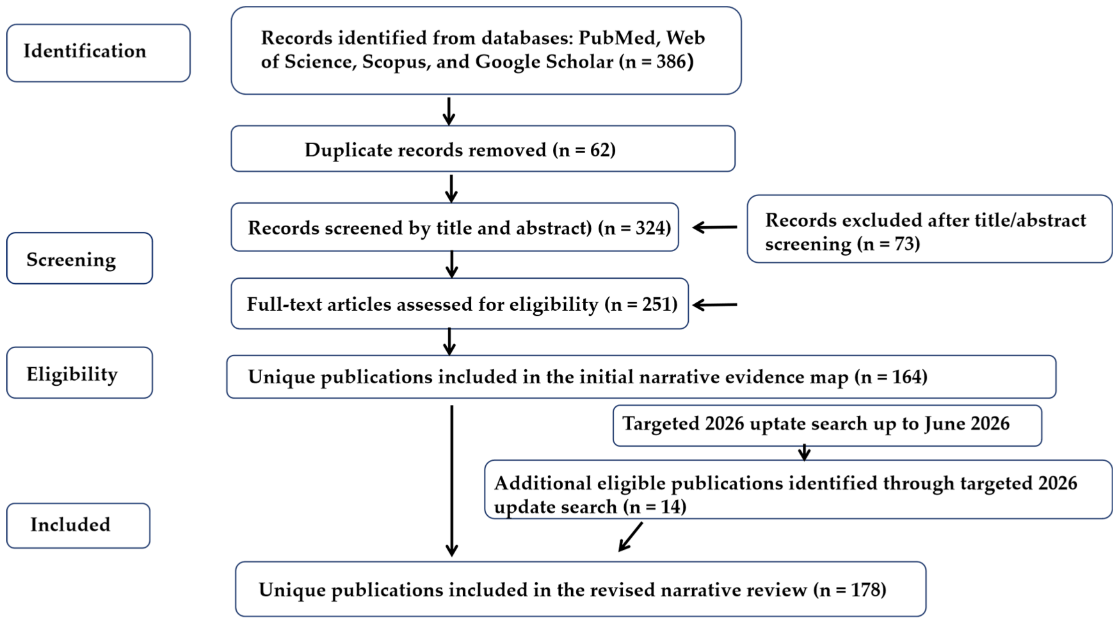

Supplementary Figure S1. Adapted PRISMA-based literature screening and targeted update workflow. A total of 386 records were initially identified from PubMed, Web of Science, Scopus, and Google Scholar. After screening and eligibility assessment, 164 unique publications were included in the initial narrative evidence map. Fourteen additional eligible publications were incorporated through a targeted update search up to June 2026. After duplicated references were counted only once, 178 unique publications were included in the revised narrative review.
